# The effect of a live-high/train-high regimen on emotional state

**DOI:** 10.1186/2046-7648-4-S1-A54

**Published:** 2015-09-14

**Authors:** Adam C McDonnell, Nektarios AM Stavrou, Ola Eiken, Igor B Mekjavic

**Affiliations:** 1 Department of Automation, Biocybernetics and Robotics, Jozef Stefan Institute, Jamova Cesta 39, 1000 Ljubljana, Slovenia; 2 Jozef Stefan International Postgraduate School, Jamova Cesta 39, 1000 Ljubljana, Slovenia; 3 Aspetar, Orthopaedic and Sports Medicine Hospital, Doha, Qatar; 4 National & Kapodistrian University of Athens, Athens, Greece; 5 Department of Environmental Physiology, Royal Institute of Technology, Stockholm, Sweden

## Introduction

We have previously reported [1] that 10 day hypoxic bedrest induces psychological strain, which is not evident during normoxic bedrest. In addition, daily ambulation while confined to a hypoxic environment also appears to prevent the hypoxic inactivity induced psychological strain. In view of the growing popularity of hypoxic training, particularly among winter athletes who live and train at altitude (Live-High/Train-High, LH/TH), we investigated the effect of such a training regimen on emotional state, as well as on the interaction among the psychological indices.

## Methods

Fourteen male participants took part in a 10-d confinement to normobaric hypoxia (P_I_O_2 _= 88.2 ± 0.6 mmHg; simulated altitude of 4175 m), conducted at the Olympic Sport Centre Planica (Rateče, Slovenia). The participants were randomly assigned either to a Live-High/Train-High group (LH/TH: two 60-minute moderate intensity exercise sessions daily on a cycle ergometer), or to a Live-High (LH) group. The participants completed the Profile of Mood States (POMS) and the Positive Affect and Negative Affect Schedule (PANAS) instruments, two days before the onset of the confinement (PRE), on the 3^rd ^(D3), 7^th ^(D7) and 10^th ^(D10) day of the confinement and on the second day of recovery (POST).

## Results

There were no significant differences revealed across the five measures in the POMS or PANAS subscales in either group (LH/TH: p = 0.325 to 0.788, LH: p = 0.345 to 0.760). High positive correlations were revealed among the negative moods (Depression, Anger, Confusion, Fatigue and Tension) during hypoxic confinement (D3: r_mean _= 0.88, D7: r_mean _= 0.81, D10: r_mean _= 0.60), while low to medium negative correlations were revealed between the positive and negative psychological indices.

## Conclusion

Hypoxic confinement induces a high correlation among the negative POMS subscales and depression. Increasing the level of daily exercise to moderate does not ameliorate this negative psychological profile.
